# Diazaphospholene and Diazaarsolene Derived Homogeneous Catalysis

**DOI:** 10.1002/chem.202001734

**Published:** 2020-07-20

**Authors:** Darren M. C. Ould, Rebecca L. Melen

**Affiliations:** ^1^ Cardiff Catalysis Institute School of Chemistry Cardiff University Main Building Cardiff CF10 3AT Cymru/Wales UK

**Keywords:** arsenic, main-group catalysis, phosphorus, reduction

## Abstract

The past 20 years has seen significant advances in main group chemistry and their use in catalysis. This Minireview showcases the recent emergence of phosphorus and arsenic containing heterocycles as catalysts. With that, we discuss how the Group 15 compounds diazaphospholenes, diazaarsolenes, and their cationic counterparts have proven to be highly effective catalysts for a wide range of reduction transformations. This Minireview highlights how the initial discovery by Gudat of the hydridic nature of the P−H bond in these systems led to these compounds being used as catalysts and discusses the wide range of examples currently present in the literature.

## Graphical abstract

**Diazaphospholenes and diazaarsolenes** are heterocyclic phosphorus and arsenic species, respectively. Recently these compounds and their corresponding cationic analogues have been used as pre‐catalysts for a wide range of reduction transformations, owing to the hydridic P‐H/ As−H bond of the active catalyst. This review discusses why these heterocycles make such good catalysts and highlights the examples currently present in the literature.

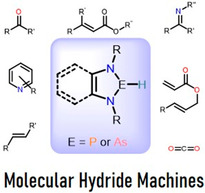

## Introduction

During the course of this century, there has been a growing surge in using main‐group compounds to replicate the roles of transition metals.^1^, ^2^ This is driven in part by the ever growing need to find more economically viable and environmentally sustainable alternatives to these metals, but also by scientific curiosity. In the past six years, diazaphospholenes (DAPs) have emerged as an interesting class of heterocycle that has proven to be effective at catalyzing a plethora of reduction‐based transformations under mild conditions. The diazaphospholene heterocycle may be simply defined as an *N*‐heterocyclic phosphine contained within a five membered unsaturated ring. Diazaphospholenes started to garner attention in the late 1990s where it was discovered that they could act as precursors to forming diazaphosphenium cations (NHPs),^3^, ^4^ which were themselves receiving significant focus.^5^

**Figure 1:**
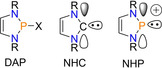
Diazaphospholene heterocycle and frontier orbital representations of *N*‐heterocyclic carbenes and diazaphosphenium cations.

Initially independently reported by both Fleming and Hutchins in 1972,^6^, ^7^ NHPs are cationic, divalent phosphorus(III) species which possess a lone pair of electrons and a vacant *p*‐orbital. These properties mean NHPs have ambiphilic character and can act as both a Lewis acid and Lewis base. However, although comparisons can be made between NHPs and the familiar Arduengo *N*‐heterocyclic carbenes (NHCs), NHPs have inverse electronic properties (Figure 1). That is, NHPs are weaker σ‐donors but much stronger π‐acceptors; a consequence of the formal positive charge and +3 oxidation state at phosphorus.^3^, ^8^, ^9^, ^10^, ^11^, ^12^

In the early 2000s the structure and reactivity of diazaphospholenes were extensively studied by Gudat, who has since reviewed this.^13^ Gudat's studies revealed that DAPs possess 6π‐delocalization in the five‐membered ring unit, but to achieve this the σ*(P–X)‐antibonding orbital is required. This in turn reduces the bond order of the P−X bond and transfers additional negative charge on the X‐atom. Thus, a compromise is reached where greater energetic stabilization in the DAP ring is achieved but at the cost of a loss of the degree of covalency in the P−X bond.^14^ It was observed that when X=H, hydridic behavior was observed, contrasted to the classically observed protic character of the hydrogen atom in the P−H bond. This was exploited by stoichiometrically reducing benzaldehyde.^15^ This observation of hydridic behavior would be key for the use of DAPs in catalytic reduction reactions. Furthermore, Gudat and colleagues reported that diazaphospholenes may be used as organocatalysts for phosphorus‐carbon bond formation from the condensation of silyl phosphine with alkyl chlorides.^16^

**Scheme 1:**
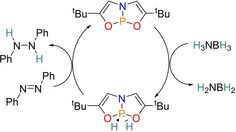
Proposed catalytic cycle for the reduction of azo benzene via P^III^/P^V^ redox cycling.

Another key discovery on the road to DAP assisted catalysis was from Radosevich and colleagues, who in 2012 first reported the reversible two‐electron redox cycling of P^III^/P^V^, which enabled it to be used for transfer hydrogenation of ammonia borane to reduce azo benzene. This was achieved by using a three‐coordinate phosphorus species with an NO_2_ type pincer ligand that forced a strained, planar T‐shaped geometry (Scheme 1).^17^

In contrast to the vast studies and attention diazaphospholenes and phosphenium cations have received, their arsenic counterparts have remained largely unexplored. The arsenic analogue of the diazaphospholene is termed diazaarsolene. Early examples of diazaarsolidines (five membered ring heterocycle containing arsenic but a saturated backbone) were reported by Wolf and Cowley,^18^, ^19^ but a literature search into diazaarsolenes gave few results. Minkin and colleagues computationally looked at the energy barrier of pyramidal inversion in diazarsolenes,^20^ but synthetic work is limited. Examples include work from Nieger et al., who synthesized 2‐halogeno‐1,3,2‐diazarsolenes,^21^ as well as reports from Gudat and Ragogna.^22^, ^23^

The first isolated and structurally characterized arsenium cations were reported by Burford in 1992,^24^ and although much rarer than phospheniums, a number of examples do exist.^19^, ^23^, ^25^, ^26^ Although a lone pair and a vacant *p*‐orbital are still present, their bonding to transition metals is typically confined to Lewis acid chemistry, where there is little to no σ‐donation from the lone pair.^27^, ^28^ This is due to the heavier pnictogen elements having a greater reluctance to form a trigonal planar geometry and so the lone pair adopts more *s*‐orbital character.^10^, ^29^

Herein, this review looks to explore the examples currently present in the literature of diazaphospholene, diazaarsolene, and their cationic counterparts in performing reduction‐based organic transformations, and to highlight the versatility these systems have. Furthermore, the catalytic cycles are discussed and mechanistic differences between the catalysts debated.

## Diazaphospholene and Diazaarsolene Assisted Reduction

### Transfer hydrogenation

**Scheme 2:**
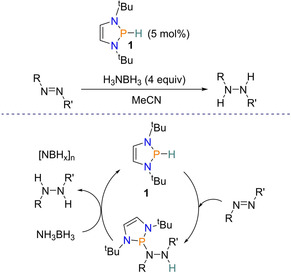
Reduction of azobenzenes with ammonia‐borane using 2‐*H*‐1,3,2‐diazaphospholene as a catalyst.

The journey to using DAPs as catalysts was first paved by the discovery of the hydridic nature of the P−H bond^15^ and the catalytic reduction of azobenzene using P^III^↔P^V^ redox cycling.^17^ These two observations led to the Kinjo group in 2014 to use 2‐*H*‐1,3,2‐diazaphospholene (**1**) for the first time as a catalyst for the reduction of azobenzenes using ammonia‐borane as the hydrogen source. After optimization, 5 mol % of the diazaphospholene **1** with four equivalents of ammonia‐borane were used for the reduction of a range of (*E*)‐azo‐compounds, giving the corresponding hydrazine product. Unlike in the P^III^↔P^V^ redox cycling case (Scheme 1), mechanistically this catalysis proceeded firstly by the addition of the P−H bond in **1** to the N=N bond to give a phosphinohydrazine. This then undergoes hydrogenolysis of the exocyclic P−N bond by hydrogen transfer from ammonia‐borane to give the desired product and regenerate **1** (Scheme 2). Investigating the mechanism further using deuterium kinetic isotope effect (DKIE) found that cleavage of the B−H and N−H bonds takes place via a concerted double cleavage pathway in the rate‐determining step.^30^

### Reduction of carbonyl groups

**Scheme 3:**
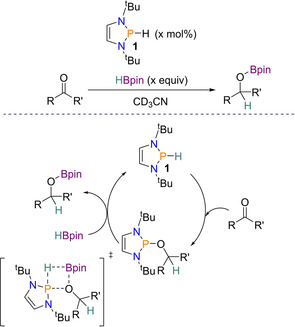
General Scheme and proposed catalytic cycle for carbonyl reduction. 0.5 mol % **1** for aldehyde reduction and 1.0 equiv HBpin; 10 mol % **1** for ketone reduction and 1.3 equiv HBpin.

Since the report on azobenezene reduction,^30^ a series of additional reductions have been reported. Although aldehydes and ketones have previously been reduced stoichiometrically by diazaphospholenes,^14^, ^31^ in 2015 this was performed catalytically in the first metal‐free catalytic hydroboration of carbonyl derivatives with pinacolborane (HBpin).^32^ Here catalytic amount of the same diazaphospholene as used for azobenezene reduction (**1**) was able to reduce aldehydes (using 0.5 mol % **1**) and ketones (using 10 mol % **1**) with HBpin (1.0 equiv and 1.3 equiv, respectively). A wide substrate scope was performed and **1** was found to be tolerant to both aliphatic and aromatic aldehydes, as well as a variety of ketones. This catalytic reaction proceeds by the formation of an alkoxyphosphine intermediate from the addition of **1** to the carbonyl substrate, where subsequent cleavage of the P−O bond and the B−H bond in HBpin gives the hydroborated product and regenerates catalyst **1**. Kinetic studies along with DFT calculations found that the bond dissociation is involved in the rate‐determining step in the transition state and that the process is stepwise, albeit almost concerted (Scheme 3).

**Scheme 4:**
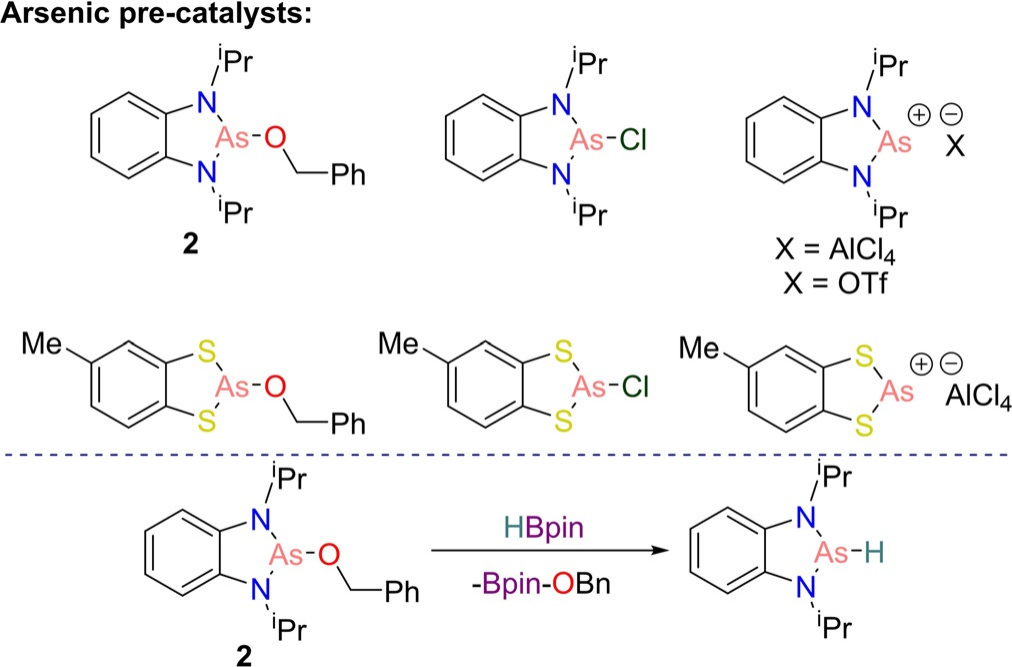
Top: Arsenic pre‐catalysts used in the optimization study. Bottom: Formation of the proposed active arsenic hydride catalyst.

Given our groups previous interest in arsenic chemistry,^33^, ^34^ we looked to determine whether arsenic could mimic this reactivity by performing hydroboration of aldehydes with HBpin. Although our systems included the fusing of a benzene ring on the backbone, recent work by Yang and Chen on the nucleophilicity of different diazaphospholenes showed that these should still be hydridic.^35^ A range of diazaarsolenes and dithiaarsolenes were synthesized, including the chloro‐ and benzyloxy‐ derivatives as well as their cations. Optimization studies found that 5 mol % of diazaarsolene **2** proved to be the most effective pre‐catalyst (Scheme 4). Proceeding with the substrate scope, **2** was shown to be an efficient pre‐catalyst for this catalysis, reducing both electron withdrawing and electron donating substrates; albeit 10 mol % catalyst loading was required for the latter. Mechanistic investigations found that the catalysis proceeds in an analogous fashion to Kinjo.^32^ The diazaarsolene pre‐catalyst reacts with HBpin to form the proposed active arsenic‐hydride catalyst (Scheme 4) via σ‐bond metathesis, where the mechanism then follows the proposed catalytic cycle shown in Scheme 3.^36^

**Scheme 5:**
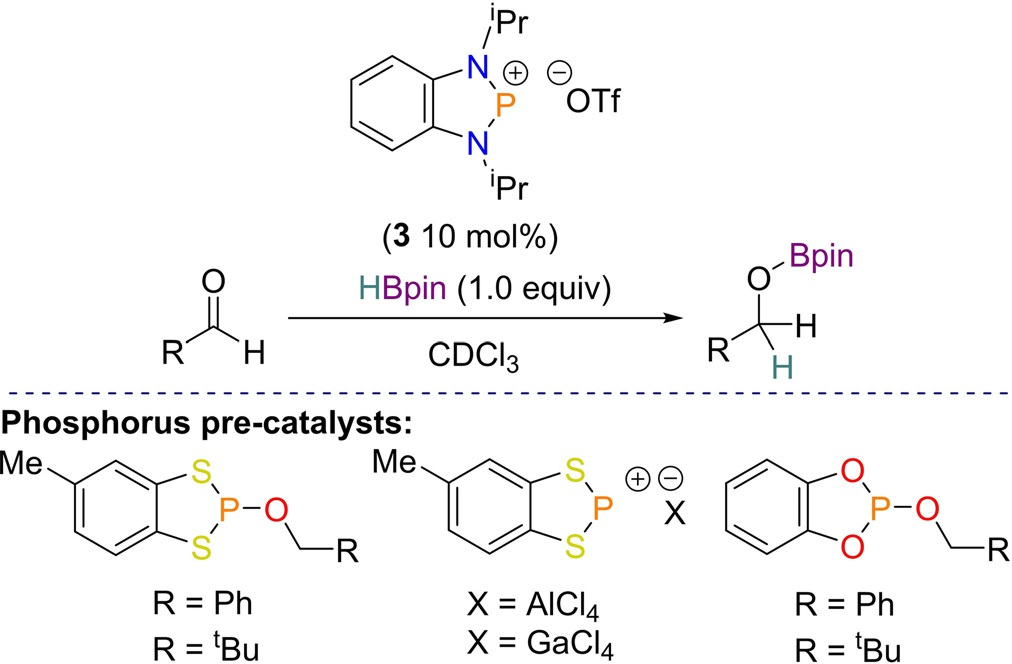
General aldehyde reduction Scheme and pre‐catalysts used in optimization study.

**Scheme 6:**
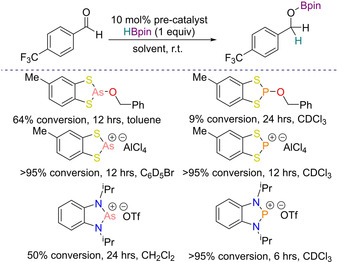
Arsenic vs. phosphorus pre‐catalyst comparison.

This reactivity was then compared to that of the phosphorus derivative. A series of diazaphospholene, dithiaphospholene, and dioxaphospholene pre‐catalysts were produced, as well as using their cationic counterparts (Scheme 5). In this case optimization reactions found that the diazaphosphenium triflate cation **3** was the best performing pre‐catalyst. Using 10 mol % **3** with one equivalent of HBpin, a series of both electron withdrawing and electron donating aldehydes were smoothly reduced. Mechanistically we proposed that this catalysis did not perform in a similar fashion to the carbonyl reduction described above,^32^ and instead involved the formation of a boronium species. However, attempts to attain mechanistic insight were thwarted by the detection of the decomposition product PH_3_ at *δ*=−238.5 ppm.^37^ From here a number of comparisons could be made between the arsenic and phosphorus systems (Scheme 6).

The neutral arsenic compounds showed greater catalytic activity than their phosphorus analogues; for example, the dithiaarsolene pre‐catalyst gave 64 % conversion of 4‐(trifluoromethyl)‐benzaldehyde to the hydroborated product after 12 hours, whereas the phosphorus analogue only achieved 9 % conversion after 24 hours in CDCl_3_. On the other hand, a less clear picture emerged from the comparison of the cationic complexes but comparing the diazaarsenium triflate to the diazaphosphenium triflate showed higher reactivity for the latter. For the former, 50 % product conversion of the hydroborated product was detected after 24 hours in CH_2_Cl_2_, whereas for the latter >95 % product conversion in CDCl_3_ was observed (Scheme 6).

### Reduction of imines

**Scheme 7:**
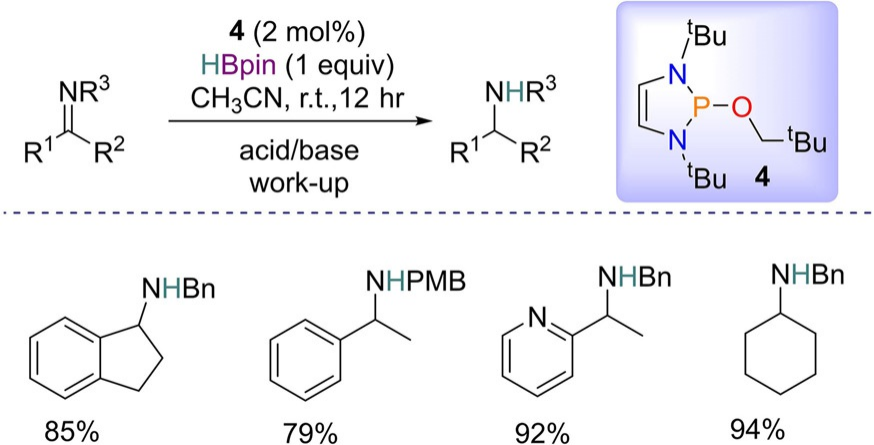
General Scheme for imine reduction and selected products.

Reduction reactions of imines is another area which has been explored using diazaphospholene based homogeneous catalysts. In 2017, Speed and colleagues looked at the reduction of imines with HBpin to produce amines (Scheme 7). A diazaphospholene similar to **1** was used in which the hydrogen atom is replaced by a neopentyloxy group (**4**). The purpose behind this was the P−H bond is sensitive to oxygen/moisture, thus the inclusion of the neopentyloxy group offers more stability to the system, making handling the diazaphospholene much more convenient for use in organic transformations. Thus **4** is a pre‐catalyst which generates the active catalyst **1** via addition of HBpin. Screening results for the optimum conditions found that 2 mol % **4** with one equivalent of HBpin at room temperature were best. Proceeding with the substrate scope, a range of imines were explored, with sterically hindered indanone‐derived imine and aldimines with different steric demand tolerated. A Lewis basic pyridyl ring was found to give no detrimental effect and, using a *p*‐methoxybenzyl (PMB) protecting group gave the expected reduced product. Aqueous (acid/base) work‐up then gave the amine product. Mechanistically, the formation of the active catalyst **1** from **4** occurs, which is then able to deliver a hydride and reduce the imine substrate.^38^

**Scheme 8:**
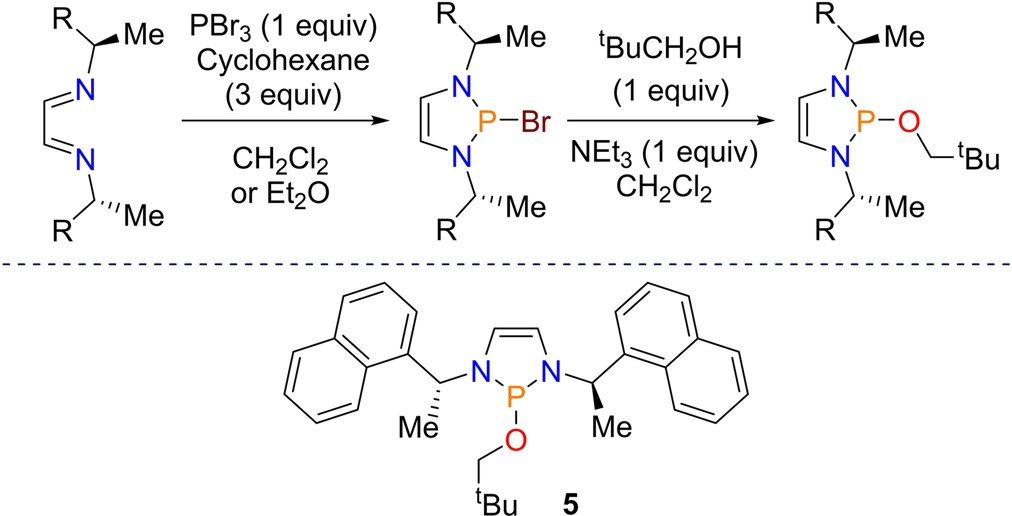
Top: General Scheme for synthesis of chiral pre‐catalyst. Bottom: Pre‐catalyst **5** used for asymmetric imine reduction.

This reduction of imines was speedily followed up by the report of the first example of enantioselective reduction using a chiral diazaphospholene. Needing a source of chirality, a chiral diimine was used. This was reacted with PBr_3_ and cyclohexene to produce a chiral diazaphospholene, bearing a P−Br bond, which was then reacted with neopentyl alcohol to produce the pre‐catalyst (Scheme 8). For the catalysis, the same optimized conditions were used as above, albeit with THF not CH_3_CN as the solvent. Asymmetric reduction of imines with HBpin was then undertaken using 2 mol % of the chiral diazaphospholene pre‐catalyst **5**. A broad substrate scope of imines flanked by aromatic groups revealed high enantiomeric ratios of up to 88:12. These results at the time were the best reported for alkyl imine hydroboration with HBpin.^39^ The mechanism for this reduction is proposed to proceed as above.

Further work on asymmetric imine reduction later led to a chiral diazaphosphenium triflate species (**6**) that could perform the catalysis. Although the use of diazaphosphenium cations as catalysts for reduction chemistry had previously been reported,^40^ this was the first example of using them for asymmetric catalysis. To make the diazaphosphenium chiral, the same ligand scaffold that was used in diazaphospholene **5** was again employed. With that, optimization reactions found that 1 mol % of diazaphosphenium **6** with 1.2 equivalents of HBpin were sufficient for the reduction. Expanding the scope, cyclic imines were found to undergo reduction, giving aryl pyrrolidines as products, with enantiomeric ratios of up to 97:3. Moreover, imines incorporating functional groups such as pyridyl rings and thiophenes, which are traditionally challenging for transition metal catalysts, were efficiently reduced.

**Scheme 9:**
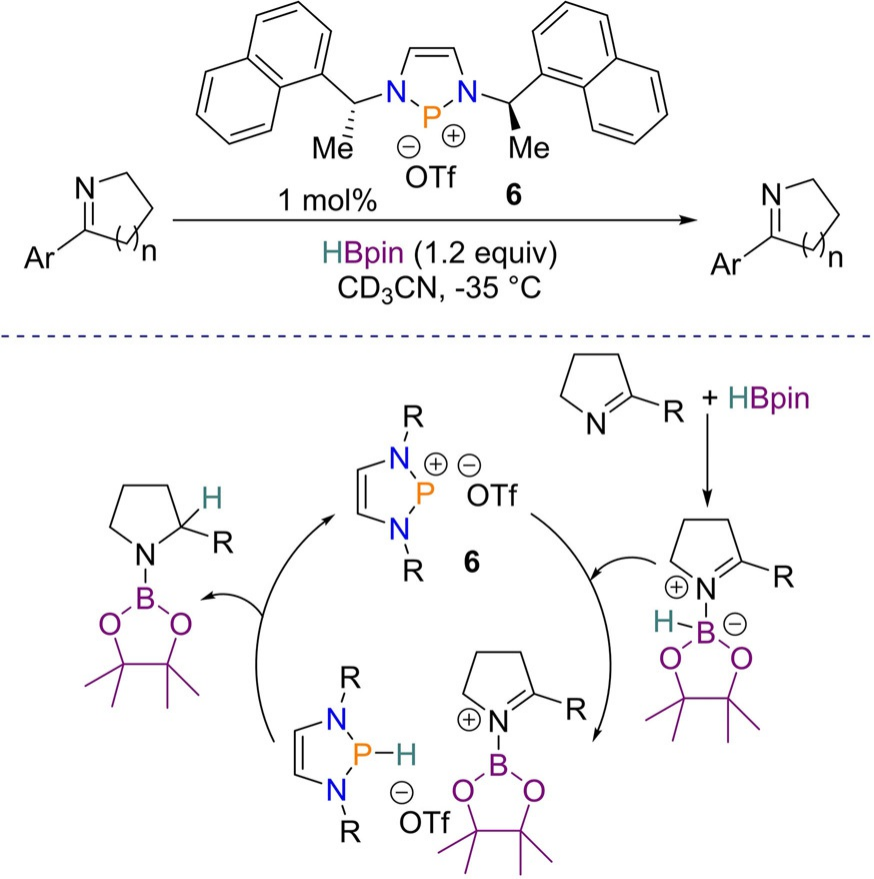
Proposed catalytic cyclic for imine reduction using a chiral diazaphosphenium cation.

Owing to the cationic nature of **6**, the mechanism is found to be dissimilar to that with diazaphospholene **4** and is proposed to be similar to other phosphenium based reduction (e.g. see reduction of pyridines later).^40^ The first step is the phosphenium cation abstracts a hydride from the activated imine‐HBpin complex, where it is then redelivered to the subsequent boranyl‐substituted iminium cation species. This gives the desired reduced imine and regenerates the catalyst. The proposed catalytic cycle is shown in Scheme 9.^41^

**Scheme 10:**
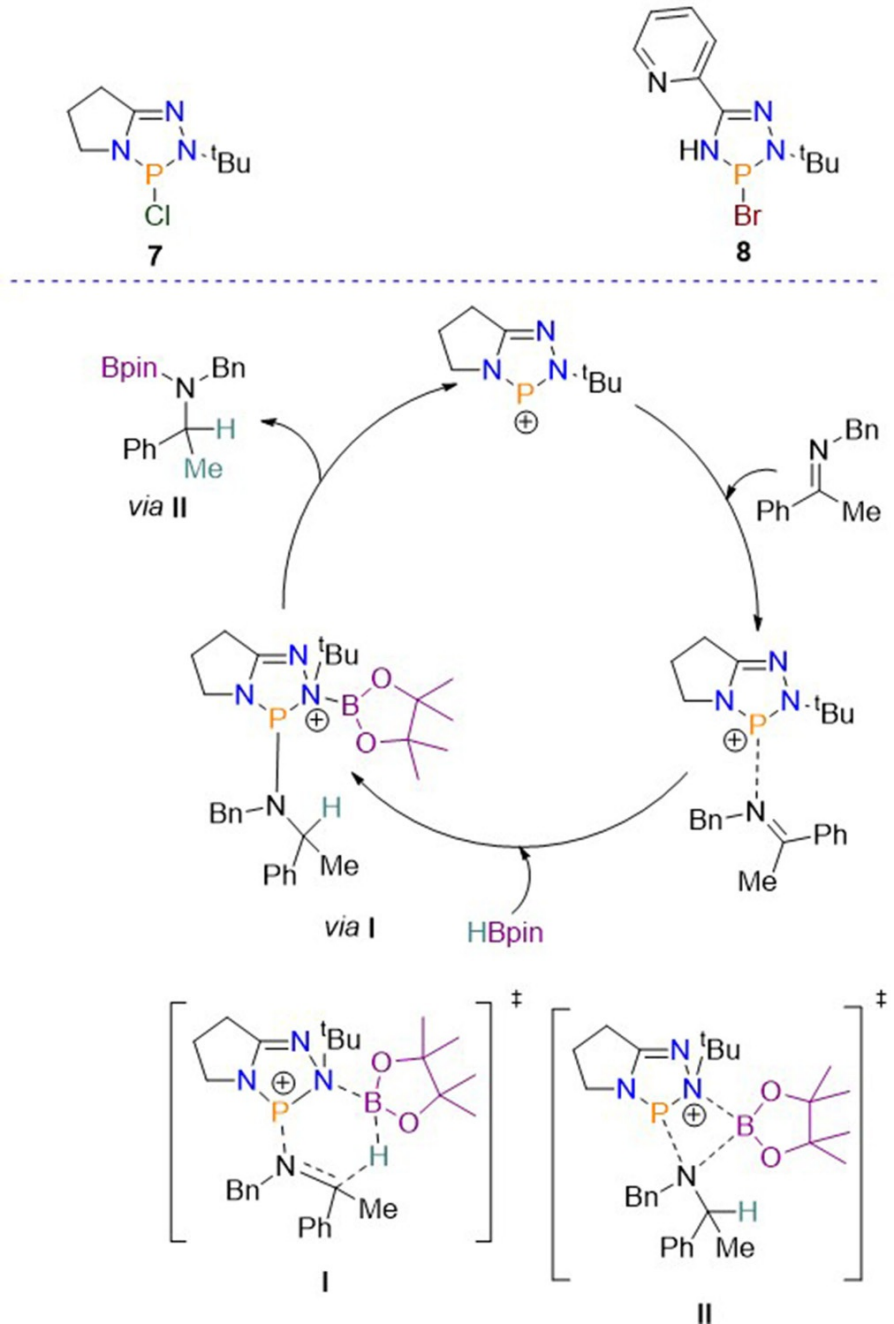
Proposed catalytic cycle for the reduction of imines using a triazaphospholene pre‐catalyst.

1,2,4,3‐triazaphospholenes have likewise been employed as catalysts. The triazaphospholene ring is similar to a diazaphospholene, except it contains three nitrogen atoms instead of two. Synthesis of the triazaphospholene pre‐catalyst is similar to diazaphospholenes but uses amidrazones as the starting ligand. Screening studies of a range of triazaphospholenes with varying steric properties found that **7** and **8** (Scheme 10) were the most suitable to proceed with a substrate scope. Using 10 mol % pre‐catalyst with 1.1 equivalents of HBpin, a variety of imines were found to undergo hydroboration readily, but more interestingly imines derived from aniline were also readily reduced. This is of interest as these substrates do not undergo reduction using diazaphospholene catalysts. Mechanistically this catalysis is intriguing since, unlike the catalytic examples discussed so far, no evidence of P−H bond formation was observed. Instead it is proposed that the pre‐catalyst is ionized in CH_3_CN, giving the cation, leading to an interaction between the positively charged phosphorus and N atom from the imine substrate. Hydride transfer via a six‐membered transition state (**I**) then occurs, after which the active catalyst is regenerated by releasing the borylated amine via **II**.^42^ The proposed catalytic cycle, as found from DFT studies, is given in Scheme 10.

**Scheme 11:**
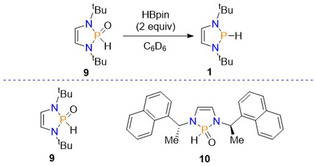
Top: Synthesis of diazaphospholene from secondary phosphine oxide. Bottom: Secondary phosphine oxides used in catalysis.

In a further attempt to develop stable main group catalysts, Speed employed air and water stable phosphine(V) oxide pre‐catalysts in the reduction of imines. In these systems the pre‐catalyst will be reduced into the catalytically active diazaphospholenes upon addition with HBpin. This work initially resulted from the observation that the diazaphosphole pre‐catalyst **4** undergoes hydrolysis to the phosphine oxide **9** over time. It was also observed that when HBpin was present, reduction of **9** to generate active catalyst **1** occurred (Scheme 11). Inspired by this, **9** was prepared from the addition of the bromide precursor to **1** and triethylamine, followed by addition of water.

**Scheme 12:**
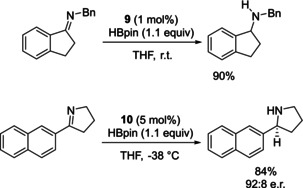
Reduction of imines using pre‐catalysts **9** and **10**.

With **9** in hand, its suitability as a pre‐catalyst was tested by performing reduction catalysis that diazaphospholenes were known to catalyze. Note that this catalysis is a variation of the above‐mentioned imine reduction with pre‐catalysts **4** and **5** (Scheme 7). With that, 1 mol % of **9** was used to catalyze the reduction of imines with 1.1 equivalents of HBpin (Scheme 12). Developing this further, enantioselective imine reduction was performed using a chiral secondary phosphine oxide (**10**). For the asymmetric catalysis, 5 mol % **10** was used, which could reduce selected imines to the corresponding amine with comparable enantioselectivity to using the chiral diazaphospholenes previously discussed (Scheme 12).^43^

### Conjugate reduction

**Scheme 13:**
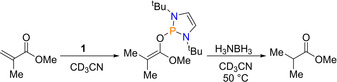
Stoichiometric addition of methyl methacrylate to diazaphospholene **1** followed by stoichiometric addition of ammonia borane.

Having previously shown that diazaphospholene **1** can promote transfer hydrogenation and reduce carbonyl bonds,^30^, ^32^ it was then shown that **1** can also enable the reduction of α,β‐unsaturated esters. To begin with, two initial stoichiometric reactions were performed: (i) reduction of methyl methacrylate using **1** to afford the 1,4‐addition product and, (ii) subsequent addition of ammonia borane to give the C=C reduced ester product (Scheme 13).

**Scheme 14:**
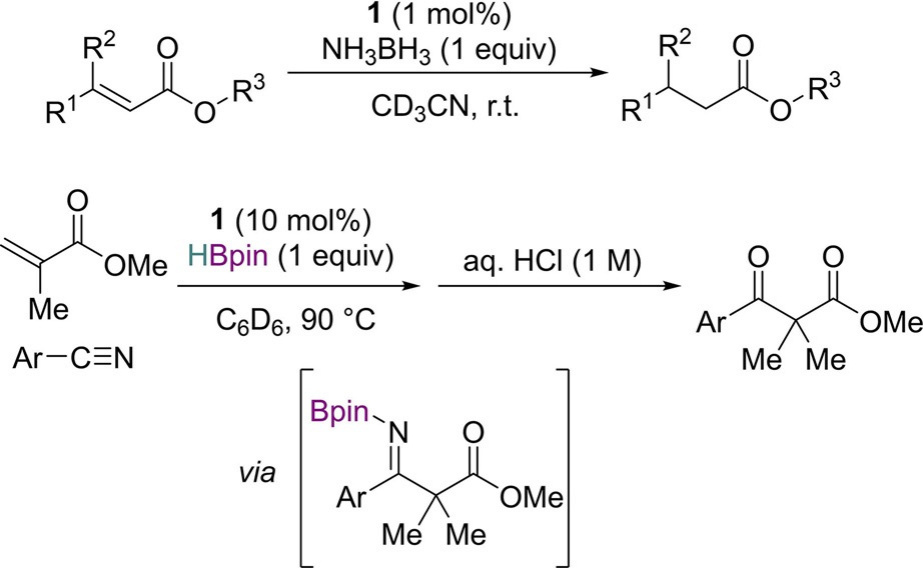
Top: Reduction of α,β‐unsaturated esters. Bottom: 1,4‐hydroboration and subsequent C−C coupling of α,β‐unsaturated esters.

Two catalytic variants based on the above stoichiometric reactions were explored, the first using ammonia borane as the reductant, affording saturated esters, and the second using HBpin to afford β‐ketoesters after a follow‐up reaction with a nitrile. In the first case, 1 mol % of **1** was used along with stoichiometric ammonia borane (Scheme 14, top). 1,4‐hydroboration of α,β‐unsaturated esters required 10 mol % **1** at 90 °C and the resulting boryl enolate intermediate was then reacted with nitriles to form the β‐ketoester product following hydrolysis (Scheme 14, bottom).

Both reactions proceed via the formation of phosphinyl enol ether from 1,4‐hydrophosphination of the α,β‐unsaturated ester (first step Scheme 13). Addition of ammonia borane then cleaves the P−O bond, generating an enol intermediate which tautomerizes to saturated esters. On the other hand, addition of HBpin again affords P−O bond cleavage, but through σ‐bond metathesis, generating a boryl enolate intermediate. This then undergoes coupling with nitriles.^44^

**Figure 2:**
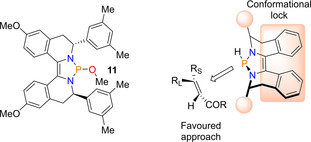
Chiral pre‐catalyst **11** and selectivity model for the asymmetric reduction.

**Scheme 15:**
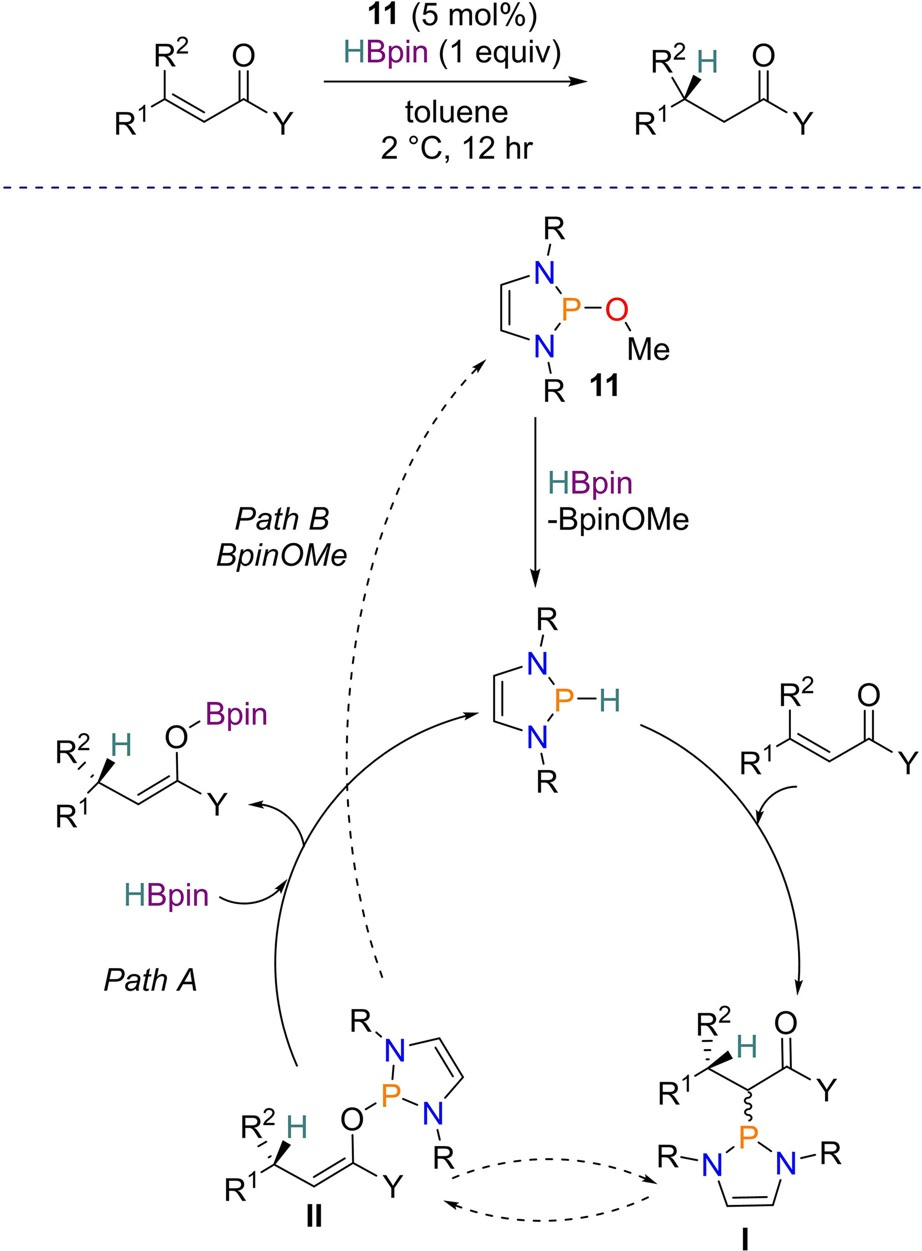
Proposed catalytic cycle for conjugate reduction with HBpin. Where Y=pyrrole fragment. Diazaphospholene shown is a simplified representation of **11**.

The Cramer group have previously had interest in the closely related diazaphospholidine heterocycle (diazaphospholene but with a saturated backbone), which they have used as ligands for metal‐based catalysis.^45^, ^46^ Therefore, given the groups interest in phosphorus heterocycles and chiral ligand design, in 2018 Cramer and colleagues reported the enantioselective conjugate reduction of α,β‐unsaturated carbonyl derivatives using diazaphospholene catalysis. To begin with, a number of chiral pre‐catalysts were synthesized, but screening results found that pre‐catalyst **11** (Figure 2), which contains 3,5‐xylyl substituents and a methoxy group in the backbone, gave the best performance for the conjugate reduction of acyl pyrrole (reaction type shown in Scheme 15). Performing a substrate scope on a range of α,β‐unsaturated acyl pyrroles using the conditions 5 mol % **11** and 1.5 equivalents of HBpin in toluene solvent gave reduced products in yields and enatiomeric ratios of up to 97 % and 95.5:4.5 respectively. In addition, chalcones were found to reduce smoothly to the corresponding ketone and the more challenging α,β‐unsaturated amides were tolerated, with an enantiomeric ratio of up to 86:14.

Upon explaining the origin of enantioselectivity in the catalysis, knowing that the P−H bond in the active catalyst is in a perpendicular position to the ring (a consequence of the pyramidal local geometry), two accessible quadrants are available away from the bulky aromatic backbone. This led to Cramer proposing the depicted stereoselectivity shown in Figure 2. Two potential catalytic cycles were proposed; Path A and Path B. In Path A, the diazaphospholene hydride is the active catalyst, where the hydride is delivered upon addition of the conjugated substrate, after which regeneration of the active catalyst occurs via addition of HBpin. This in turn gives a boron enolate, which after hydrolytic work‐up generates the final product. Alternatively, in Path B the first part of the catalytic cycle is the same, but the coordinated intermediate **II** undergoes σ‐bond metathesis with pinBOMe (produced from the earlier σ‐bond metathesis step), regenerating **11** and giving the boron enolate (Scheme 15).^47^ Note Path A is the same as that reported with catalyst **1**.^44^

**Scheme 16:**
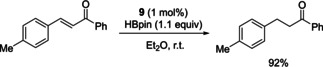
Conjugate reduction using pre‐catalyst **9**.

Phosphine oxide pre‐catalyst **9** could also enable conjugate reduction, where chalcone was smoothly reduced using 1 mol % of **9** and 1.1 equivalents of HBpin (Scheme 16).

### Reductive Claisen rearrangement

**Scheme 17:**
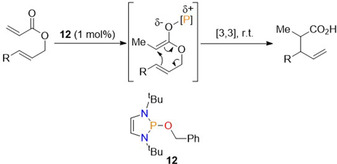
General Scheme for diazaphospholene catalyzed reductive Claisen rearrangement.

Following the report of conjugate reduction of α,β‐unsaturated carbonyl derivatives, the use of the benzyloxy derived diazaphospholene **12** as a pre‐catalyst for the reductive Claisen rearrangement was reported (Scheme 17).^48^ This is a transformation in which a [3,3]‐sigmatropic rearrangement converts allyl vinyl ethers to unsaturated carbonyl species.

**Scheme 18:**
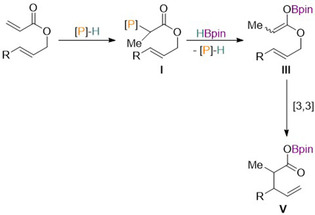
Proposed first mechanistic pathway for reductive Claisen rearrangement. [P]‐H=**1**.

Using catalytic diazaphospholene **12** for this transformation, the optimization studies exposed allyl 2‐phenylacrylate to an array of terminal reductants, where HBpin proved most effective for the transformation in combination with 1 mol % **12**. A substrate scope followed, where a wide array of allylic acrylates bearing various functional groups were found to be tolerated for the rearrangement, which was also enantiospecific for substrates with existing stereogenic centers. Investigations into the diasteroselectivity found it could be tuned by varying the solvent as well as changing the diazaphospholene catalyst, suggesting several pathways exist depending on the nature of the pre‐catalyst and substrate. Thus, two possible mechanisms are proposed for the reaction. In the first proposed pathway (Scheme 18), the addition of the active catalyst **1** gives intermediate **I**, which reacts with HBpin to form boron enolate **III** via σ‐bond metathesis. In turn, intermediate **III** rearranges to **V**.

**Scheme 19:**
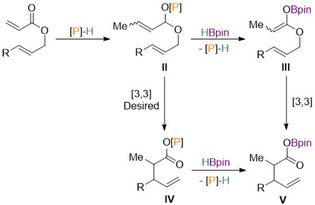
Proposed second mechanistic pathway for reductive Claisen rearrangement. [P]‐H=**1**.

On the other hand, a second mechanistic pathway may take place (Scheme 19), where addition of the active catalyst gives intermediate **II**. From here two options are possible and both involve a [3,3]‐sigmatropic rearrangement and elimination of catalyst **1** and differ only in their ordering. Intermediate **II** forming intermediate **IV** is most desirable as this would allow greater control of the diastereoselectivity and enantioselectivity by the bound diazaphospholene.

### Reduction of pyridines

**Scheme 20:**
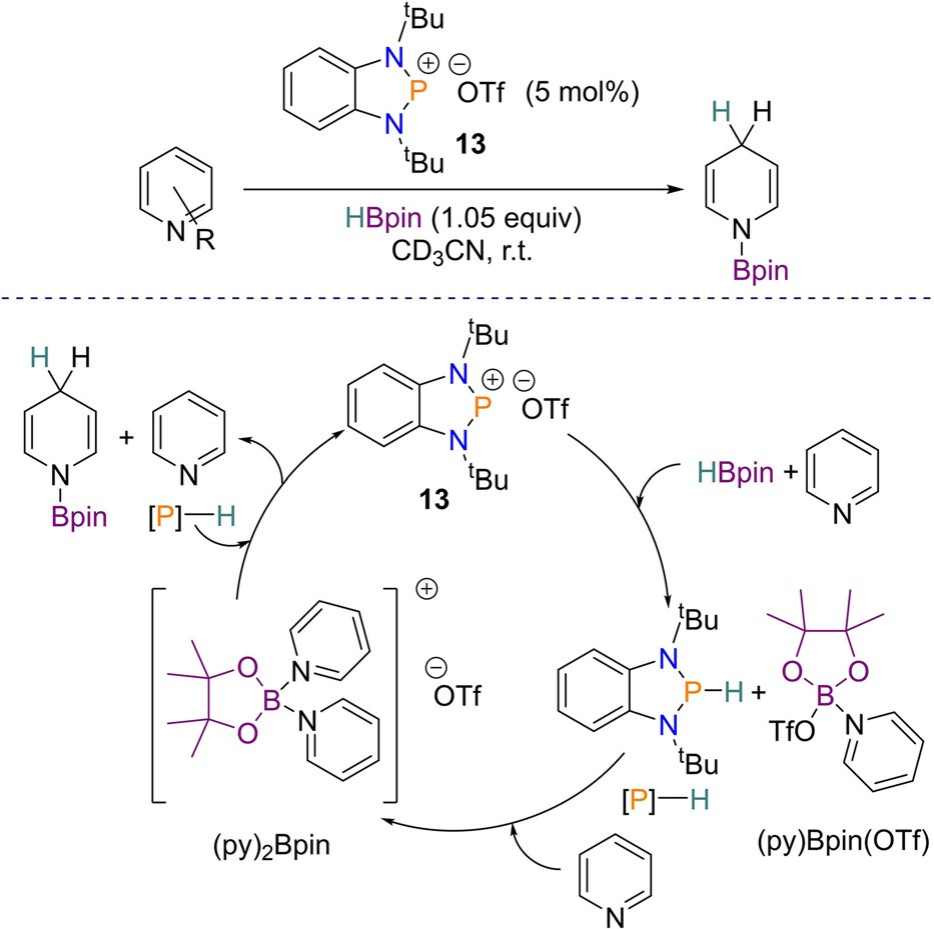
Proposed catalytic cycle for pyridine reduction using diazaphosphenium **13** as a pre‐catalyst.

Dihydropyridines are commonly found in biological molecules such as NADH (nicotinamide adenine dinucleotide) and are also useful in synthetic chemistry (e.g. Hantzsch esters). Their synthesis from the corresponding pyridines is nevertheless challenging owing to the stability of the aromatic ring and usually preactivated systems are required. In 2018, diazaphosphenium cations were found to serve as an effective pre‐catalyst for the reduction of pyridines with HBpin. After a series of screening reactions with different cations of varying steric properties, the diazaphosphenium **13** proved to be the most effective pre‐catalyst to proceed with. Along with 1.05 equivalents of HBpin, 5 mol % **13** was used for the substrate scope, where a variety of substituted pyridines were found to be smoothly reduced with both regio‐ and chemo‐selectivity. Good functional group tolerance was observed when the pyridine ring was substituted in the *meta*‐position, however substitution in the *ortho*‐ and *para*‐position proved more challenging. Given the cationic nature of **13** this catalysis does not proceed in an analogous fashion to that with the neutral diazaphospholene **1**. Instead, investigations found that the first step involves hydride transfer from HBpin to **13**, generating diazaphosphenium‐hydride and the boronium salt [(py)_2_Bpin]OTf. The second step is then reduction of the activated pyridine via hydride delivery from the diazaphosphenium‐hydride (Scheme 20).^40^

**Scheme 21:**
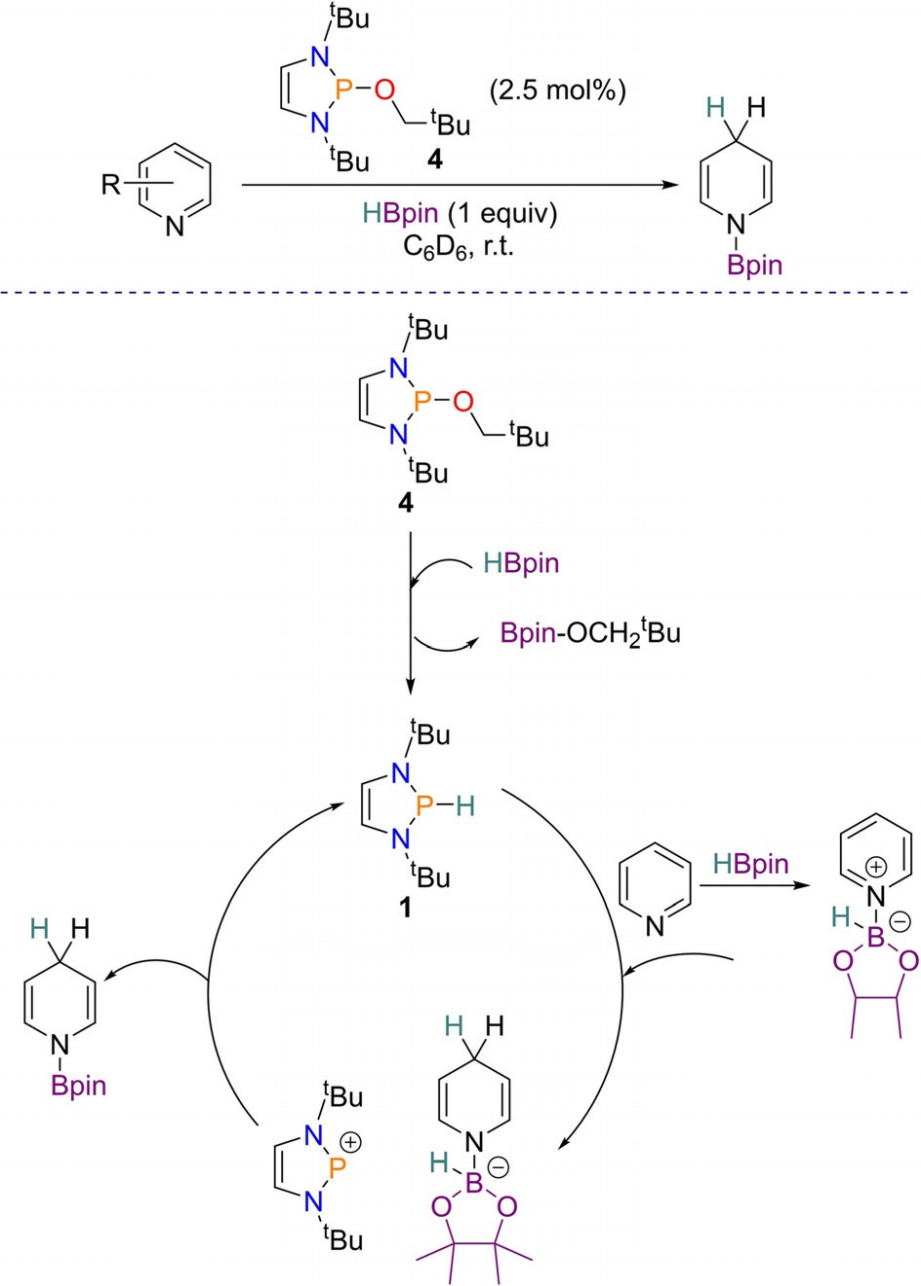
Proposed catalytic cycle for pyridine reduction using diazaphospholene **4** as a pre‐catalyst.

It was found that neutral diazaphospholenes can also be used for this reduction, with 2.5 mol % pre‐catalyst **4** effective for reducing pyridines with HBpin (1 equiv). Substrates bearing electron‐withdrawing groups in the *meta*‐position worked well, but again *ortho*‐ and *para*‐substituted pyridines were more challenging. Mechanistically this pyridine reduction is different to the example reported above. The first step is postulated to be formation of the active catalyst **1** via σ‐bond metathesis, after which pyridine reduction takes place from hydride delivery. From here B−P hydride transfer is speculated to occur, giving the desired hydroborated pyridine product and regenerating catalyst **1** (Scheme 21).

Comparing reductions of neutral diazaphospholenes with cationic diazaphospheniums shows that the latter is able to tolerate more electron rich pyridines, whereas the former requires more electron withdrawing groups attached to the pyridine ring for smooth reduction to take place. On the other hand, the diazaphospholene pre‐catalyst operates well in low polarity solvents (such as [D_6_]benzene), whereas the diazaphosphenium cation does not.^49^

**Scheme 22:**
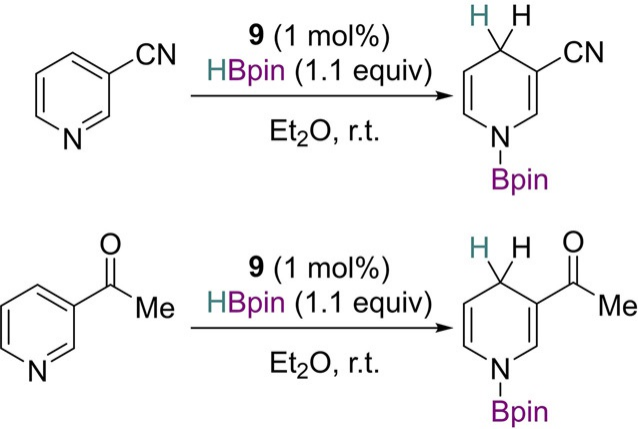
Pyridine reduction using **9** as a pre‐catalyst.

Finally, the phosphine oxide pre‐catalyst **9** described earlier was also used for pyridine reduction, which when using 1 mol % pre‐catalyst with 1.1 equivalents of HBpin, niconitrile was found to be effectively reduced. Interestingly, 3‐acetylpyridine was selectively reduced, with the ketone moiety remaining untouched (Scheme 22).^43^

### Reduction of CO_2_

**Scheme 23:**
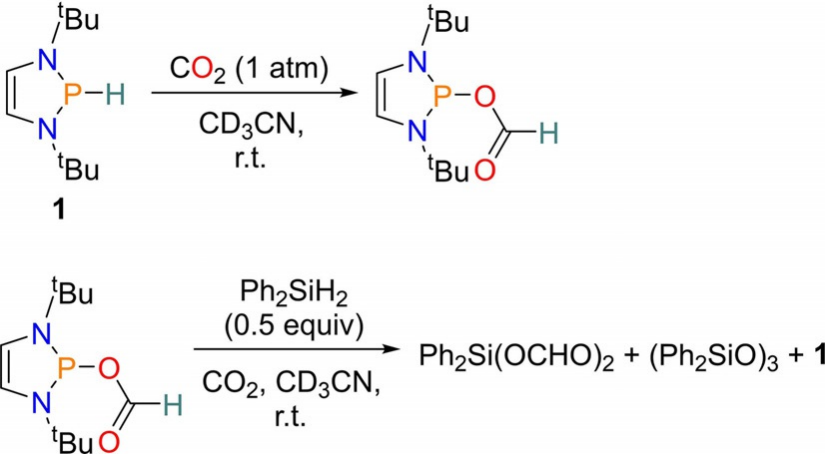
Top: Hydrophosphination of CO_2_ with **1**. Bottom: Formate transfer with Ph_2_SiH_2_ to regenerate **1**.

The use of CO_2_ as a C1 source is potentially very powerful as it offers a non‐toxic way to build more synthetically useful products in a cheap manner, but also gives a use for this harmful greenhouse gas.^50^ As a result of this, the catalytic reduction of CO_2_ has been investigated using the diazaphospholene catalyst **1**. The diazaphospholene was found to undergo a hydrophosphination reaction with CO_2_ (1 atm), producing a diazaphospholene species with a formate group attached (Scheme 23, top). This transformation is a consequence of the oxygen group from CO_2_ inserting into the P−H bond of **1** along with hydride transfer to the carbon atom of CO_2_. It was postulated that the formate group should readily transfer to an acceptor. Thus, the formate intermediate was reacted with half an equivalent of Ph_2_SiH_2_. Ph_2_Si(OCHO)_2_ resulted as the major product and the siloxane (Ph_2_SiO)_3_ as a minor product (2.3:1 respectively) (Scheme 23, bottom). Moreover, it was later discovered that the formate transfer step can be accelerated by adding 5 mol % **1**.

**Scheme 24:**
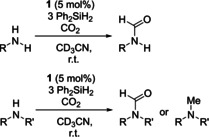
Top: Catalytic *N*‐formylation primary. Bottom: secondary amines with CO_2_ using 5 mol % **1** as a catalyst.

Subsequently, the one‐pot *N*‐formylation of amines with CO_2_, using 5 mol % **1** as a catalyst (Scheme 24) was performed. For the catalysis, a wide substrate scope of both primary and secondary amines was used. For the secondary amines, less‐hindered aliphatic amines gave the *N*‐formylamine in excellent yields of >90 %, but an increase in sterics afforded *N*‐methylated amines. Secondary amines containing aryl substituents were found to be tolerated. Expanding the scope, all aliphatic and aromatic primary amines tested were found to work well, with yields in the range of 72 % to 99 %.^51^

## Conclusions and Outlook

In this Minireview, the use of the heterocyclic diazaphospholenes, diazaarsolenes and their cationic counterparts as catalysts for organic reduction transformations has been evaluated. In 2014, the catalytic reduction of azobenzene using 2‐*H*‐1,3,2‐diazaphospholene was first reported, making use of the hydridic P−H bond these complexes possess. Since then the reduction of carbonyls, imines, α,β‐unsaturated esters, pyridines and CO_2_ have all been reported. In these cases a number of diazaphospholene species have been utilized, with the use of an alkoxide derived co‐ligand providing an advancement in the field due to increased moisture/oxygen tolerance compared to 2‐*H*‐1,3,2‐diazaphospholene. Further advances have come from the inclusion of a chiral ligand scaffold allowing for enatioselective catalysis. Halide abstraction from diazaphospholenes results in cationic phosphenium formation, and these cations have proved to be highly effective for these reductions, and in certain cases outperforming the neutral diazaphospholene. In addition to this, heavier Group 15 arsenic pre‐catalysts have been developed, including diazaarsolenes and diazaarsenium cations. However, in general, the reactivity and tolerance were diminished compared to the phosphorus counterparts.

Although several similar mechanisms operate in these reactions, a key feature is the formation of a P−H bond in the catalytic cycle. Importantly, the hydridic nature of the P−H bond opens the possibility for these phosphorus containing heterocycles to be used for a vast array of reduction reactions. We are only at the beginning of the field and it is likely that many more catalysts and differing reactivity will be uncovered in the near future.

## Conflict of interest

The authors declare no conflict of interest.

## Biographical Information

*Dr. Darren M. C. Ould undertook his undergraduate studies at Cardiff University, where he received his integrated Masters degree (MChem) in 2016. Following this, he undertook a PhD position under the supervision of Dr. Rebecca L. Melen at Cardiff University. His main research interest was on the synthesis and reactivity of novel phosphorus and arsenic heterocycles*.

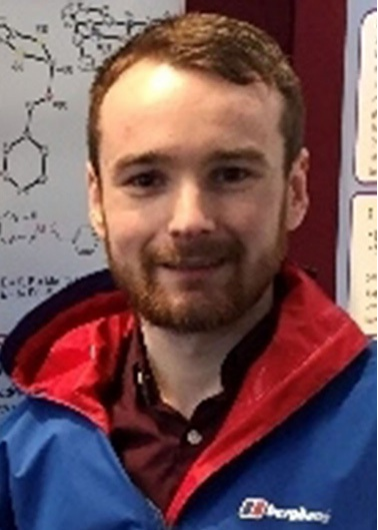

## Biographical Information

*Dr. Rebecca L. Melen studied for her undergraduate and PhD degrees at the University of Cambridge, completing her PhD in 2012 with Prof. Wright. Following postdoctoral studies with Prof. Stephen in Toronto and with Prof. Gade in Heidelberg, she took up a position at Cardiff University in 2014, where she is now a Reader. In 2018, she was awarded an ESPRC fellowship and she is the 2019 recipient of the RSC Harrison Meldola Memorial Prize. Her research interests lie in main group chemistry and the use of main group Lewis acids in synthesis and catalysis*.

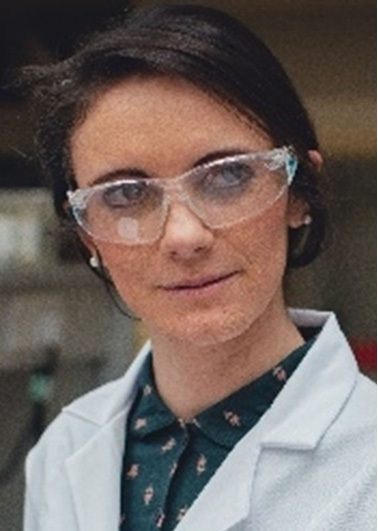

## References

[1] P. P. Power , Nature 2010, 463, 171–177. 20075912 10.1038/nature08634

[2] C. Weetman , S. Inoue , ChemCatChem 2018, 10, 4213–4228.

[3] D. Gudat , Eur. J. Inorg. Chem. 1998, 1087–1094.

[4] M. K. Denk , S. Gupta , A. J. Lough , Eur. J. Inorg. Chem. 1999, 41–49.

[5] A. H. Cowley , R. A. Kemp , Chem. Rev. 1985, 85, 367–382.

[6] S. Fleming , M. K. Lupton , K. Jekot , Inorg. Chem. 1972, 11, 2534–2540.

[7] B. E. Maryanoff , R. O. Hutchins , J. Org. Chem. 1972, 37, 3475–3480.

[8] M. B. Abrams , B. L. Scott , R. T. Baker , Organometallics 2000, 19, 4944–4956.

[9] D. Gudat , Top. Heterocycl. Chem. 2010, 21, 63–102.

[10] H. M. Tuononen , R. Roesler , J. L. Dutton , P. J. Ragogna , Inorg. Chem. 2007, 46, 10693–10706. 17997547 10.1021/ic701350e

[11] L. Rosenberg , Coord. Chem. Rev. 2012, 256, 606–626.

[12] D. Gudat , Comprehensive Inorg. Chem. II, 2013, vol. 1, 587–621.

[13] D. Gudat , Dalton Trans. 2016, 45, 5896–5907. 26863391 10.1039/c6dt00085a

[14] D. Gudat , A. Haghverdi , H. Hupfer , M. Nieger , Chem. Eur. J. 2000, 6, 3414–3425. 11039535 10.1002/1521-3765(20000915)6:18<3414::aid-chem3414>3.0.co;2-p

[15] D. Gudat , A. Haghverdi , M. Nieger , Angew. Chem. Int. Ed. 2000, 39, 3084–3086; 11028040

[16] S. Burck , D. Förster , D. Gudat , Chem. Commun. 2006, 2810–2812. 10.1039/b605278a 17009471

[17] N. L. Dunn , M. Ha , A. T. Radosevich , J. Am. Chem. Soc. 2012, 134, 11330–11333. 22746974 10.1021/ja302963p

[18] C. Payrastre , Y. Madaule , J. G. Wolf , Tetrahedron Lett. 1990, 31, 1145–1146.

[19] C. J. Carmalt , V. Lomeli , B. G. McBurnett , A. H. Cowley , Chem. Commun. 1997, 2095–2096.

[20] E. G. Nesterova , R. M. Minyaev , V. I. Minkin , Russ. J. Org. Chem. 2003, 39, 1167–1173.

[21] T. Gans-Eichler , D. Gudat , M. Nieger , Heteroat. Chem. 2005, 16, 327–338.

[22] S. Burck , M. Nieger , D. Gudat , Z. Anorg. Allg. Chem. 2010, 636, 1263–1267.

[23] J. T. Price , M. Lui , N. D. Jones , P. J. Ragogna , Inorg. Chem. 2011, 50, 12810–12817. 22092146 10.1021/ic201983n

[24] N. Burford , T. M. Parks , B. W. Royan , B. Borecka , T. S. Cameron , J. F. Richardson , E. J. Gabe , R. Hynes , J. Am. Chem. Soc. 1992, 114, 8147–8153.

[25] N. Burford , T. M. Parks , P. K. Bakshi , T. S. Cameron , Angew. Chem. Int. Ed. Engl. 1994, 33, 1267–1268;

[26] K. Izod , P. Evans , P. G. Waddell , Angew. Chem. Int. Ed. 2019, 58, 11007–11012; 10.1002/anie.201905922 31157950

[27] K. A. Porter , A. C. Willis , J. Zank , S. B. Wild , Inorg. Chem. 2002, 41, 6380–6386. 12444781 10.1021/ic020410j

[28] N. Burford , J. A. C. Clyburne , P. Losier , T. M. Parks , T. S. Cameron , J. F. Richardson , Phosphorus Sulfur and Silicon 1994, 93–94, 301–304.

[29] S. Burck , J. Daniels , T. Gans-Eichler , D. Gudat , K. Nättinen , M. Nieger , Z. Anorg. Allg. Chem. 2005, 631, 1403–1412.

[30] C. C. Chong , H. Hirao , R. Kinjo , Angew. Chem. Int. Ed. 2014, 53, 3342–3346; 10.1002/anie.201400099 24615812

[31] S. Burck , D. Gudat , M. Nieger , W.-W. Du Mont , J. Am. Chem. Soc. 2006, 128, 3946–3955. 16551102 10.1021/ja057827j

[32] C. C. Chong , H. Hirao , R. Kinjo , Angew. Chem. Int. Ed. 2015, 54, 190–194; 10.1002/anie.201408760 25376892

[33] T. T. P. Tran , D. M. C. Ould , L. C. Wilkins , D. S. Wright , R. L. Melen , J. M. Rawson , CrystEngComm 2017, 19, 4696–4699.

[34] D. M. C. Ould , A. C. Rigby , L. C. Wilkins , S. J. Adams , J. A. Platts , S. J. A. Pope , E. Richards , R. L. Melen , Organometallics 2018, 37, 712–719.

[35] J. Zhang , J. D. Yang , J. P. Cheng , Angew. Chem. Int. Ed. 2019, 58, 5983–5987; 10.1002/anie.201901456 30805968

[36] D. M. C. Ould , R. L. Melen , Chem. Eur. J. 2018, 24, 15201–15204. 30088671 10.1002/chem.201803508

[37] D. M. C. Ould , T. T. P. Tran , J. M. Rawson , R. L. Melen , Dalton Trans. 2019, 48, 16922–16935. 31687708 10.1039/c9dt03577j

[38] M. R. Adams , C. H. Tien , B. S. N. Huchenski , M. J. Ferguson , A. W. H. Speed , Angew. Chem. Int. Ed. 2017, 56, 6268–6271; 10.1002/anie.201611570 28145614

[39] M. R. Adams , C. H. Tien , R. McDonald , A. W. H. Speed , Angew. Chem. Int. Ed. 2017, 56, 16660–16663; 10.1002/anie.201709926 29115745

[40] B. Rao , C. C. Chong , R. Kinjo , J. Am. Chem. Soc. 2018, 140, 652–656. 29303259 10.1021/jacs.7b09754

[41] T. Lundrigan , E. N. Welsh , T. Hynes , C. H. Tien , M. R. Adams , K. R. Roy , K. N. Robertson , A. W. H. Speed , J. Am. Chem. Soc. 2019, 141, 14083–14088. 31441650 10.1021/jacs.9b07293

[42] C. H. Tien , M. R. Adams , M. J. Ferguson , E. R. Johnson , A. W. H. Speed , Org. Lett. 2017, 19, 5565–5568. 28994602 10.1021/acs.orglett.7b02695

[43] T. Lundrigan , C.-H. Tien , K. N. Robertson , A. W. H. Speed , Chem. Commun. 2020, 10.1039/d0cc01072c. 32159538

[44] C. C. Chong , B. Rao , R. Kinjo , ACS Catal. 2017, 7, 5814–5819.

[45] P. A. Donets , N. Cramer , J. Am. Chem. Soc. 2013, 135, 11772–11775. 23909575 10.1021/ja406730t

[46] J. Pedroni , N. Cramer , J. Am. Chem. Soc. 2017, 139, 12398–12401. 28832139 10.1021/jacs.7b07024

[47] S. Miaskiewicz , J. H. Reed , P. A. Donets , C. C. Oliveira , N. Cramer , Angew. Chem. Int. Ed. 2018, 57, 4039–4042; 10.1002/anie.201801300 29461670

[48] J. H. Reed , P. A. Donets , S. Miaskiewicz , N. Cramer , Angew. Chem. Int. Ed. 2019, 58, 8893–8897; 10.1002/anie.201904411 31044498

[49] T. Hynes , E. N. Welsh , R. McDonald , M. J. Ferguson , A. W. H. Speed , Organometallics 2018, 37, 841–844.

[50] D. M. D′Alessandro , B. Smit , J. R. Long , Angew. Chem. Int. Ed. 2010, 49, 6058–6082; 10.1002/anie.201000431 20652916

[51] C. C. Chong , R. Kinjo , Angew. Chem. Int. Ed. 2015, 54, 12116–12120; 10.1002/anie.201505244 26276547

